# Dental-derived mesenchymal stem cell sheets: a prospective tissue engineering for regenerative medicine

**DOI:** 10.1186/s13287-022-02716-3

**Published:** 2022-01-29

**Authors:** Yuanting Chen, Huacong Huang, Gaoxing Li, Jianyu Yu, Fuchun Fang, Wei Qiu

**Affiliations:** 1 Department of Stomatology, Nanfang Hospital, Southern Medical University, 1838 Guangzhou Avenue North, Guangzhou, 510515 People’s Republic of China; 2 School of Stomatology, Southern Medical University, 1838 Guangzhou Avenue North, Guangzhou, 510515 People’s Republic of China

**Keywords:** Dental-derived mesenchymal stem cells, Cell sheets, Decellularized, Regenerative medicine

## Abstract

Stem cells transplantation is the main method of tissue engineering regeneration treatment, the viability and therapeutic efficiency are limited. Scaffold materials also play an important role in tissue engineering, whereas there are still many limitations, such as rejection and toxic side effects caused by scaffold materials. Cell sheet engineering is a scaffold-free tissue technology, which avoids the side effects of traditional scaffolds and maximizes the function of stem cells. It is increasingly being used in the field of tissue regenerative medicine. Dental-derived mesenchymal stem cells (DMSCs) are multipotent cells that exist in various dental tissues and can be used in stem cell-based therapy, which is impactful in regenerative medicine. Emerging evidences show that cell sheets derived from DMSCs have better effects in the field of regenerative medicine applications. Extracellular matrix (ECM) is the main component of cell sheets, which is a dynamic repository of signalling biological molecules and has a variety of biological functions and may play an important role in the application of cell sheets. In this review, we summarized the application status, mechanisms that sheets and ECM may play and future prospect of DMSC sheets on regeneration medicine.

## Introduction

DMSCs are multipotent cells that can be found in various dental tissues and have the characteristics of mesenchymal cells (MSCs). DMSCs include human dental pulp stem cells (DPSCs) [1], stem cells from human exfoliated deciduous teeth (SHED) [2], periodontal ligament stem cells (PDLSCs) [3], stem cells from the apical papilla (SCAPs) [4], gingival mesenchymal stem cells (GMSCs) [5], dental follicle stem cells (DFSCs) [6], tooth germ stem cells (TGSCs) [7] and alveolar bone-derived mesenchymal stem cells (ABMSCs) [8]. Most of them are derived from the neural crest, and have the potential of multipotent differentiation. For example, as research found that they can differentiate into adipocytes, osteocytes, chondrocytes, neurocytes, hepatocytes and pancreocytes [9]. In previous work, DMSCs presented immunomodulatory properties by secreting cytokines and developing immune receptors [10] and showed great potential capability of controlling odontogenic differentiation [11]. DMSCs are easy to culture, as they can be obtained from a wide range of sources, such as deciduous teeth, extracted orthodontic teeth or third molars, with minor damage on subjects and no ethical controversy. All above-mentioned characteristics of DMSCs make them stand out from other mesenchymal stem cells, regarding stem cell-based therapy, which is of great importance in regenerative medicine. DMSCs have been applied in some preclinical studies and clinical trials as a treatment of dental and neurodegenerative diseases, spinal cord injury, eye disease, immune diseases, orthopaedic disorders and so forth [12].

Stem cells therapy plays a therapeutic role through the three major mechanisms of stem cells differentiation, paracrine and homing. However, the limitation in cell number and getting lost easily become the major shortcomings of stem cells injection during the process. Cell sheet technology, firstly invented by Okano [13], is a unique cell delivery method with high engraftment efficiency and targeting. This technology forms the high-density cells sheet-like structures that contain only cells and their secreted ECM, which preserve cell–matrix connections, intercellular connections and specific shape provided by ECM. ECM is mainly composed of two types of secreted macromolecules: fibrous proteins and glycoproteins. These cell-associated proteins provide a certain support strength level and space filling function, regulating protein complexes, participating in cell signal transduction, and promoting cell adhesion [14]. As a scaffold-free structure, cell sheets provide a three-dimensional structure for cell proliferation and differentiation and help avoid the defects of scaffold materials that may lead to tissue inflammation or toxic reactions. In addition, cell sheets protect cells when they are digested by enzymes, which is necessary in scaffold technology [15].

In recent years, scientists have applied certain decellularized treatments to cell sheets. Decellularization technology refers to the effective removal of cellular and nuclear components, especially DNA and RNA, while retaining the essential components, biological activity and mechanical integrity of the decellularized extracellular matrix (dECM). Due to the lack of cells and the major histocompatibility complexes in dECM, the inflammatory reaction is significantly reduced, or even eliminated, in response to foreign bodies and immune rejection, while biological activity, biochemical composition, original 3D structure and mechanical integrity are highly preserved [14]. Consequently, the utilization of decellularization in regenerative medicine is on the rise. Moreover, DMSC sheets are combined with cytokines or new materials for better tissue regeneration effects, which has been utilized in many tissue regeneration processes, such as dental tissue regeneration, bone regeneration, revascularization, facial nerve regeneration and corneal regeneration.

**Fig. 1:**
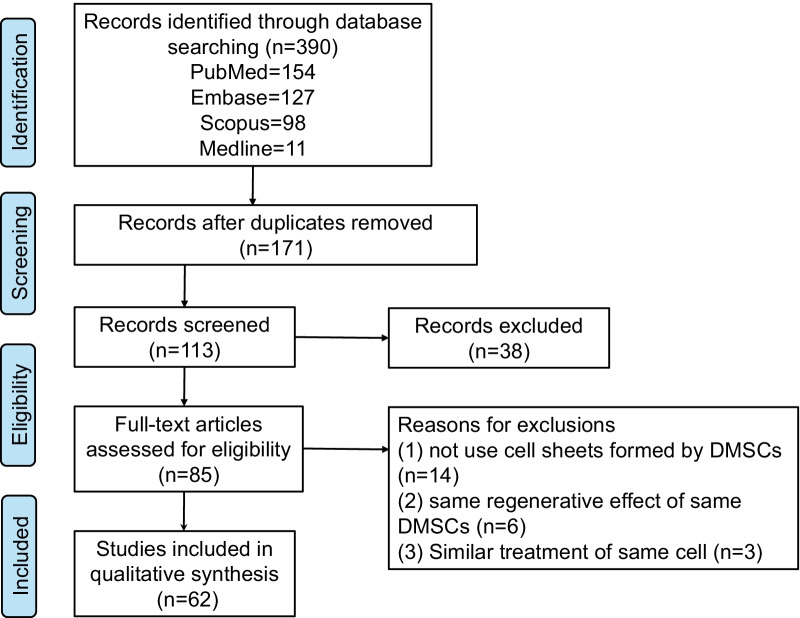
Schematic flowchart representing study inclusion

In this review, systematic literature search was conducted for 4 databases including PubMed, Embase, Scopus, Medline. The applied search terms were broad, including synonyms, truncations and synonyms of ‘dental-derived stem cell’, ‘cell sheet’, ‘regenerative medicine’. Schematic flowchart representing study inclusion is shown in Fig. 1. Additionally, the application status, mechanisms of cell sheets and ECM, future prospect of DMSC sheets on regeneration medicine were summarized as well.

## Stem cell sheets

According to existing research, stem cell sheets take the dominant place in cell sheets regenerative therapies and have been applied in periodontal ligament [16], myocardial tissue [17], cornea [18], and liver tissue [19] regeneration. During the culture and separation process, the activity and structure of the stem cell should be preserved, as well as ECM, so as to maximize the function of the cell sheets. Various methods have been developed to produce fully functioning regenerative cell sheets that can be modified or directly to different tissues.

### Stem cell sheets preparation

There are three common methods to fabricate cell sheets using different surface culture dishes, namely, temperature-responsive polymer, other surface modification, as well as non-surface modification methods. First of all, the temperature-responsive polymer approach is to apply temperature-reactive polymer treatment on the surface of cell culture dishes, in which the adhesion and separation of cells are changed in different temperatures. Second, other surface modification techniques involve the application of gold-plated coatings, photoreactive materials and materials inducing reactive oxygen species (ROS) generation on the surface of the culture medium. The mature cell sheets are separated from the medium surface by an electro-reaction cell membrane separation system, light reaction system and green light-emitting diode (LED) irradiation. Lastly, non-surface modification techniques include lysozyme treatment, fibrin matrix coating, magnetic nanoparticles, and ultrasonic irradiation methods for separating cell sheets [20].

**Fig. 2:**
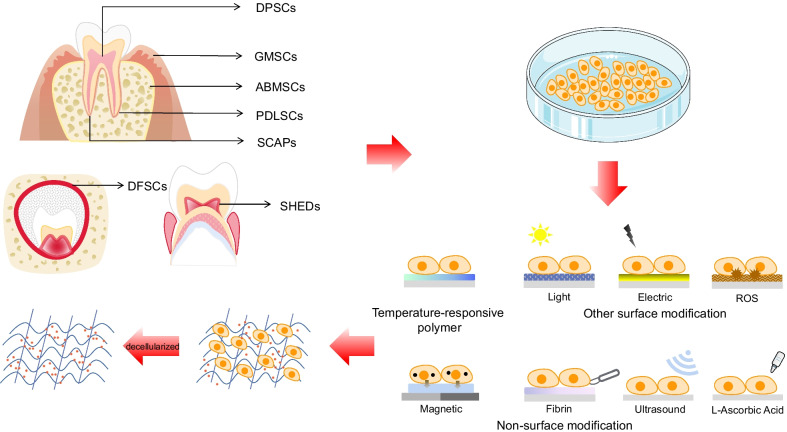
The origins, cell sheets preparation and decellularization of dental-derived mesenchymal stem cells (DMSCs). Dental-derived mesenchymal stem cells (DMSCs) consist of dental pulp stem cells (DPSCs), stem cells from human exfoliated deciduous teeth (SHED), periodontal ligament stem cells (PDLSCs), dental papilla stem cells (SCAPs), gingival mesenchymal stem cells (GMSCs), dental follicle stem cells (DFSCs), tooth germ stem cells (TGSCs) and alveolar bone-derived mesenchymal stem cells (ABMSCs). There are four common ways to harvest mature cell sheets: temperature-responsive polymer; other surface modification; non-surface modification methods; and adding a certain concentration of L-Ascorbic acid to the cell culture medium. The decellularized extracellular matrix (dECM) was obtained after processing to remove cells

Nowadays, the most common method to synthesize DMSC sheets is to add a certain concentration of L-Ascorbic acid to the cell culture medium, since it boosts cell sheet formation by increasing the amount of ECM. After 7–14 days, the DMSC sheets can be harvested when the shrinkage at the bottom edge of the culture medium [21]. The combination of L-Ascorbic acid and temperature-responsive culture dishes is a popular method for cell sheet culture [22], as shown in Fig. 2. Beside DMSCs, the majority of bone marrow mesenchymal stem cells (BMSCs), adipose-derived stem cells (ADSCs), umbilical cord mesenchymal stem cells (UC-MSCs) and other MSC sheets are also produced through this method [23].

### Decellularization treatment

Due to the fact that decellularization is affected by two main factors: the methods of decellularization and tissue or cell characteristics, the decellularization scheme needs to be selected according to the specific tissue or cell characteristics, for the purpose of removing cell components, maintaining the natural ECM characteristics. All the following treatments are able to trigger decellularization: chemical, physical, enzymatic treatments and additional agents, in which chemical method uses least time. As for chemical methods, ionic or non-ionic surfactants, acids and bases, ethanol and other solvents are used to dissolve cells and decompose DNA and RNA, SDS and Triton X-100, for instance. On the other hand, enzymatic treatments usually require enzymes, such as nucleases (deoxyribonuclease and trypsin), to digest cell components. In physical treatments, the most common methods are temperature, pressure, electroporation and impact/force of mechanical agitation, slicing or mincing cells. Additional agents include tributyl phosphate (TBP), chelating agents, toxins, and supercritical carbon dioxide [14, 24, 25].

## DPSC sheets applications

As a new scaffold-free material, DPSC sheets have been used in dental tissue, bone, nerve and corneal regeneration. Derived from DPSCs, DPSC sheets contain much more vascular formations, and the fibres are more porous than other dental stem cell sheets, according to previous study [26]. The single DPSC sheet was directly applied to tissue defects at the outset, with the addition of growth factors to the cell sheets during the culture process. Improved techniques were invented later on to obtain a better regeneration effect and meet the morphological needs of a variety of defects. Photobiomodulation therapy (PBMT) is the fourth element of tissue engineering, which enhances the effectiveness when applied with stem cells, growth factors, and scaffolds. PBMT is capable of increasing the fibronectin level of the ECM in DPSC sheets, resulting in tighter bonding between cells, as well as between cells and the ECM. Additionally, it probably helps the maturation of ECM, therefore increasing malleability of ECM during transplantation [21].

### Dental tissue regeneration of DPSC sheets

Dental tissue regeneration can be categorized as the regeneration of dental pulp, alveolar bone, periodontal soft tissue and tooth root. As we all know, endodontic disease is one of the most common dental disease categories. Unfortunately, the current clinical treatments are likely to cause tooth reinfection or fracture. In recent years, increasing attention has been paid to the treatment of pulp regeneration of pulpless teeth. For example, the popularity of DMSCs application to mediate pulp regeneration is on the rise and validated animal models and clinical trials are also developed. Sufficient blood supply in the root canal is important during the pulp regeneration process, and therefore the vascular regeneration of the graft is a prerequisite for pulp regeneration. DPSC sheets were made into a rod-like shape, which is suitable for transplantation into root canals for a maximizing regeneration function. The three-dimensional rod-like structure enables sufficient oxygen and nutrients for DPSCs. The results from previous study showed that the outer DPSCs differentiated into odontoblast-like mineralizing cells, and pulp-like tissue, containing abundant blood vessels, was observed in the full root canal, which was similar to natural dental pulp. Conversely, no blood components were found in teeth without DPSCs constructs. Additionally, it is likely that most DPSCs within the structure had higher survival possibility, because the greater amount of ECM fibres and proteoglycan content enabled the culture medium to infiltrate better. Therefore, DPSCs had better access to oxygen, nutrients and inhibit cell apoptosis [27]. The dental pulp regeneration produced by biomimetic material with self-forming ability is a successful example in dental regenerative medicine and this material is expected to be applied in more regenerative areas closely related to angiogenesis in the near future.

Periodontitis causes progressive destruction of the periodontal support tissue, including alveolar bone resorption, attachment loss, and matrix destruction [28]. To uncover the effect of DPSCs on the treatment of periodontal regeneration, DPSCs or sheets were directly transplanted to periodontal defects in minipigs. The results presented that although new bone regeneration was observed in all groups, the bone mass of the DPSC sheets group was significantly higher than the counterpart in DPSCs injection group, which suggested a greater impact of DPSC sheets on periodontal tissue regeneration [29]. DPSCs apoptosis decreased in hypoxic environment or serum-free medium while blood vessel regeneration increased when hepatocyte growth factor (HGF) gene was transfected. Notably, both the DPSCs and DPSC-HGF injection contributed to periodontal alveolar bone regeneration and soft tissue healing, but the alveolar bone volume and the percentage of bone tissue in the periodontium in the DPSC sheets group were higher [30]. Existing preclinical studies showed that DPSCs and DPSC-HGF sheets were able to significantly improve periodontal bone regeneration, but the underlying mechanisms and longer-term effects need to be further investigated.

Tooth loss is another common dental disease. Nowadays, biologically inactive dental implants have become the most commonly used method for teeth restoration [31]. There is no periodontal connection built between implants and alveolar bone, leading to disability of distributing chewing pressure and failed feeling changes in temperature or pressure because of a lack of pulp tissue. One regenerative strategy uses stem cells, graft materials, and cell-signalling molecules mimicking the interlayer structure of natural teeth, which contains periodontal tissue, dentin, and pulp, to regenerate the ideal root. Periodontal tissue-like fibres were found on the lateral side of TDM, a large amount of predentin like tissue was found on the pulp side of TDM 12 weeks after transplantation with DPSC sheets, treated with dentin matrix (TDM) and matrigel, and odontoblast like cells were found near and inside the regenerated predentin as well. TDM can release dentin sialophosphoprotein (DSPP), dentin matrix protein 1 (DMP-1), transforming growth factor-β (TGF-β) and other growth factors to create a favourable induced microenvironment for root regeneration. Upregulation of vascular endothelial growth factor receptor 1 (VEGFR1) and Nestin, CD31 and β3-tubulin in regenerative pulp-like tissue indicated that the composite structure with the DPSC sheets can promote pulp and bone regeneration [32]. In summary, there might be a new way of root regeneration in the future.

Recently, decellularized tissues have been applied to regenerative medicine, including human peripheral nerves, skeletal muscle and cardiac tissue. The regeneration function of decellularized pulp tissues has drawn wide attention. For example, a study demonstrated that DPSCs seeded onto decellularized dental pulp showed 3-dimensional attachment and proliferation, in which cell differentiation was observed near the dentinal wall. Increased expression of odontoblastic markers and cellular projections into dentinal tubules posed a positive influence on endodontic regeneration [33]. In another similar study, ECM harvested from decellularized pulp tissues was implanted into the root canals of beagle dogs for 8 weeks. At the end, CD31-positive and dentin sialoprotein (DSP)-positive tissues were found in the root canals, indicating the formation of neovascularization and dental pulp-like tissue, respectively. In addition, mineralized tissue was formed in the root canal, which was attributed to the differentiation of DPSCs into cementoblasts. It is widely believed that cementogenesis inhibitors are needed to prevent mineralization in the root canal during pulp regeneration. However, dental pulp tissue ECM has a positive and reliable effect on tissue reconstruction and site-appropriate tissue regeneration, which is likely attributed to dECM providing cells a niche environment, including unique pattern of distribution and functional components [34]. At the present, researches on DMSCs dECM were limited; however, it may become a new direction of stem cells therapy soon due to its excellent regenerative potential, multifunction and flexibility as a biomaterial, as well as making up for immune rejection and shortage of sources.

### Bone regeneration of DPSC sheets

Bone defects can be caused by a variety of diseases, injuries, or congenital developmental abnormalities, which are usually treated by autologous or allogeneic bone grafts. However, limited donor bone, secondary surgical trauma, simultaneous immunosuppressive therapy, an elevated risk of infection from bacteria or viruses and postoperative instability can also lead to problems during the treatment. Hence, an effective yet safe regenerative treatment for bone defects is expected. Based on the osteogenic differentiation potential of DPSCs, researchers have attempted to apply DPSC sheets to bone fractures in order to promote bone tissue growth. 4-(4-Methoxyphenyl) pyrido [40,30:4,5] thieno [2,3-b] pyridine-2-carboxamide (TH) is a helioxanthin derivative. After the transplantation of the TH-treated DPSC sheets into the skull defect, bone regeneration was significantly promoted, and the DPSC sheets were covering defects throughout the entire repair period [22, 35]. It has been demonstrated that DPSC sheets have higher immunosuppressive activity and better ability to suppress T cell responses compared to BMSCs. Aging causes different changes in various tissues and organs, such as volume reduction in dental pulp tissue, resulting in the number of DPSCs collected from the lost teeth of elderly patients is less than that obtained from young patients. However, as for the process of promoting bone formation, no significant difference was found when comparing DPSCs from young and older individuals. What is interesting is that DPSCs from elderly individuals still show a strong bone regeneration ability because the telomere length remained stable from P1 to P3. It is possible that once DPSCs obtained ‘stemness’ after passing a specific stage, the proliferation and differentiation potential are independent from the aging process [36]. As a consequence, the age of the donor will not be a major limiting factor in future clinical applications. Three-dimensional cell culture refers to the co-culture of scaffolds and cells with different three-dimensional structures in vitro that could establish the physiological interaction between cells and the cell-extracellular matrix to simulate the specificity of natural tissue to the greatest extent [37]. Using three-dimensional cell culture technology, the single DPSC sheet was constructed into a spherical tissue-engineered structure, which promoted the differentiation of DPSCs into mature osteoblasts, displayed the related gene expression of osteoblasts, collagen type 1 (Col 1) was expressed widely from the surface layer of the tissue structure to its centre and induced the accumulation of a large amount of bone matrix protein and calcified matrix grafting to the bone defect site to promote bone regeneration. In the regeneration process, COL 1 acts as a scaffold on which a calcified matrix is deposited, eventually forming bone tissue [38].

### Nervous system regeneration of DPSC sheets

Attributed to be of neural crest origin, DPSCs show remarkable neurogenic potential and have been applied to many neurodegenerative diseases and nerve injuries, including Alzheimer's disease, Parkinson’s disease and spinal cord injury. However, the number of transplanted DPSCs is much lower than anticipated [39]. Therefore, the DPSC sheets may be a better choice to reach ideal nerve generation. Facial nerve injury is a type of peripheral nerve injury. The proportion of axon regeneration is cogent evidence of nerve function repair. After transplanted DPSC sheets to facial nerve, they can stay in the injured site for a long time and produce a large amount of neurotrophic factor (NTF) protein, including brain-derived neurotrophic factor (BDNF), glial cell-derived neurotrophic factor (GDNF) and neurotrophic factor-3 (NT3), which have been demonstrated to have roles in peripheral nerve development and repair. DPSC sheets act as an NTF transmission system to promote axon extension and significantly enhance nerve regeneration. All these results suggest the optimistic outlook of DPSC sheets for the treatment of peripheral nerve injury [40]. Currently, new biomaterials, such as nerve conduits and porous scaffolds, are widely used in the treatment of peripheral nerve injury and spinal cord injury. Upon binding with bFGF, chitosan scaffolds activated the extracellular signal-regulated kinase (ERK) pathway and enhanced the expression of neural markers in DPSCs [41, 42]. Heparin-poloxamer (HP), a thermosensitive material that transforms into hydrogel structure at body temperature, improved the motor function recovery of a rat spinal cord injury model and reduced the release of pro-inflammatory factors around the injury sites after grafted in vivo combined with DPSCs [43, 44]. Nerve conduits, which are made from biodegradable materials, have become reliable clinical scaffold materials for peripheral nerve injury treatment. In combination of recombinant human basic fibroblast growth factor and DPSCs, cells differentiated into neurons and Schwann-like cells and formed myelinated nerve fibres, which confirmed that the conduit could promote nerve repair and regeneration in vivo [45]. The combination of these scaffold materials and cell sheets to provide bioactive components by ECM has been preliminarily explored, and it is believed that this is a treatment method that can be expected to achieve better nerve regeneration effects in future.

In addition, DPSC sheets are also applicable to other tissue regeneration treatments. In an animal model of total limbal stem cell deficiency (LSCD), DPSC sheets enabled better functional regeneration and transparency of damaged corneas in mice and expressed limbal stem cell markers in in vitro culture [46]. To summarize, DPSC sheets had been proved to be effective in the regeneration of dental tissue, bone tissue, nerve tissue and cornea. The regenerated tissue from DPSC sheets contained more angiogenesis and the fibrous structure was more porous, similar to loose connective tissue, indicating that they are more suitable for regeneration of dental pulp or vascular rich tissues. In addition, DPSC seems to show stronger stemness during the cell sheets formation. The expression of related genes to self-renewing and stemness maintenance of mesenchymal stem cells in DPSC sheets is also higher than that in other DMSC sheets [26], suggesting that DPSC sheets are likely to differentiate into multi-line tissues under certain conditions. Therefore, according to the characteristics of DPSC and the existing experimental results, DPSC can achieve the regeneration of a variety of tissues, but it is especially suitable for loose tissues containing blood vessels. Currently, clinical studies have not been performed, and the regeneration and potential side effects of DPSC sheets in human need to be further explored.

## PDLSC sheets applications

PDLSCs are mesenchymal-derived stem cells isolated from the periodontal ligament. It has been previously confirmed that PDLSCs have the potential for cementum and osteogenic differentiation [3], which can form a three-dimensional periodontal ligament tissue-like structure [47], and have the potential to differentiate into endothelial cells, cardiomyocytes and retinal ganglia-like cells [48]. Because of the multilineage differentiation potential, PDLSC sheets have been used in periodontal tissue, bone tissue, and corneal regeneration. In the future, a wider application of PDLSC sheets treating diseases is expected.

### Periodontal tissue regeneration of PDLSC sheets

Periodontal tissue incorporates cementum, periodontal ligament and alveolar bone. This complex structure characterized by bone and ligament connects soft and hard tissues functionally and endows natural teeth with physiological functions, such as reducing excessive occlusal force, carrying out corrective movement through bone remodelling and perceiving harmful stimulation. PDLSCs exhibit osteoblast-like characteristics and are able to differentiate into osteoblasts or osteoblasts. They are also considered as the optimal seed cells for periodontal regeneration [49].

A biphasic scaffold is formed by solution electrospinning and melt electrospinning, and then the cell sheets are combined to form the final multiphasic construct. An important feature of this construct is that it ensures sufficient biomechanical stability to maintain close contact between the cell sheet and the root surface, thereby facilitating attachment to the root surface. It was found that the multiphasic construct with PDLSC sheets could achieve satisfying periodontal tissue regeneration. Although regenerated periodontal tissue contained implanted PDLSCs, most of the cells involved in the formation of mature regenerated tissue were from surrounding host tissue [50]. However, the implantation of biphasic scaffolds with primary GMSC sheets inhibited the recovery of periodontal tissue. This might be because GMSCs primary culture consists of a heterogeneous population of terminally differentiated fibroblasts and progenitor cells, which are not beneficial to periodontal regeneration [51]. Titania nanotubular topography has been widely proven to be a powerful regulator of cell shape, adhesion, proliferation and differentiation. However, it has never been reported whether the function of PDLSCs can be modulated by nanotopography [52, 53]. The nanomorphology of the titanium surface, after surface treatment, can provide cues for periodontal regeneration and enhance the adhesion and diffusion of PDLSCs and the secretion of collagen in the initial stage after the implantation of sheets and significantly improved the PDLSCs differentiation into periodontal ligament (PDL) cells in the sheets, generation of PDL-like dense collagen fibres and vascular regeneration also occurred [54]. In addition to using the cell sheets in combination with other scaffolds, the use of PDLSC sheets to self-form a cylindrical structure also achieved excellent regeneration effects. After the cultured PDLSC sheets formed a cylindrical structure, the scaffold-free structure formed a PDL-like fibrous structure outside and mineralized tissue inside. In addition, the PDL-like structure in vitro was parallel to the cementum. When exposed to the mechanical stimulation microenvironment of the natural periodontal tissue, the arrangement of cells and ECM in the tissue changed to some extent, suggesting that moderate mechanical stimulation is important for the structure and morphology of the periodontal tissue [55]. A previous study suggested that individual PDLSCs transduced with recombinant human bone morphogenetic protein-2 (rhBMP-2) promoted the formation of mineralized tissue [56]. In another study, rhBMP-2 pretreatment promoted collagen fibre formation from PDLSC sheets and cementogenic differentiation. Cementum-like mineralized tissue with vertical or oblique insertion of collagen fibres onto the micro-/macro-porous biphasic calcium phosphate (MBCP) structure surface were formed [57]. Nano morphology, self-formed structure or additional inducers, these treatment factors facilitated the tissue regeneration from different perspectives and mechanisms. To explore the cell sheets with fewer side effects and better regeneration effects, pre-treated DMSC, combining with other biological scaffold materials, or additional treatment can be taken into consideration.

With the wide application of titanium implants in the restoration of missing teeth, increasing attention has been given to peri-implant inflammation. Without timely intervention, it will lead to rapid progression of inflammation, bone destruction, and ultimately failure of implant surgery [31]. One of the strategies of peri-implantitis regeneration therapy is cell sheet technology. PDL-like fibrous tissue was observed on the surface of the implant after the titanium rod wrapped with PDLSC sheets were implanted into the jaw defect. The calcium phosphate coating has been proven to accelerate the calcification of osteoblasts, and the coating can improve the attachment of PDL cells to the surface of titanium. An important function of the periodontal ligament is to buffer the occlusal load and maintain alveolar bone height. This important physiological function is missing due to the titanium implant and jawbone direct combination. Therefore, the ability to generate periodontal tissue around the implant with the cell sheets technique is of great significance to dental health [58]. Most experiments with cell sheets have been conducted at the cellular level and in animal models, but the regenerative effect of PDLSC sheets have been validated in patients with periodontitis. The availability of autologous suitable, redundant healthy periodontal tissue is often a limiting factor for periodontitis patients; however, owing to the immunomodulatory role of MSCs, suitable allogenic tissue may also be a reliable source [16]. Decellularized periodontal ligament ECM retains its original structure, effectively supports the regeneration of pericementum PDLSCs, and expresses cementum- and periodontal ligament-related genes after reseeding PDLSCs onto ECM [59]. In summary, the integration of autologous MSCs with a decellularized periodontal scaffold may be a promising approach to promote PDL regeneration.

### Bone regeneration of PDLSC sheets

The potential for osteogenic differentiation of PDLSCs has been demonstrated by the formation of calcified nodules and the expression of alkaline phosphatase (ALP), matrix extracellular protein, bone sialoprotein (BSP), osteocalcin (OCN) and TGF-β receptor I [3]. Recently, a variety of treatments have been applied to improve the regeneration effect of PDLSC sheets, including the addition of active molecules during the culture of cell sheets, modification of scaffold structure and gene transfection. Previous studies have demonstrated that platelet-rich fibrin (PRF) can promote the regeneration of oral tissues. During the fabrication of PDLSC sheets, PRF treatment promoted the proliferation of PDLSCs and induced osteogenic differentiation by increasing the expression of the runt-related transcription factor 2 (RUNX2), BSP, osteopontin (OPN), and OCN genes [60]. Osthole is a small molecular compound extracted from traditional Chinese medicine that has been reported to promote the proliferation and differentiation of osteoblasts [61, 62]. After treatment with Osthole, the levels of osteogenic-related genes and proteins in PDLSC sheets were significantly improved, and more bone formation was observed in vivo [63]. Gold nanoparticles (AuNPs) are novel biocompatible materials, and AuNPs have been reported to promote the osteogenic differentiation of PDLSCs in a particle size-dependent manner [64, 65]. When AuNPs were added to PDLSC sheets, the CT, bone staining and bone formation-related protein results showed more new bone formation [66].

Indirectly co-cultured technology is another way to promote bone regenerative potential of PDLSC sheets. It has been suggested that co-culture with jawbone marrow-derived mesenchymal stem cells, endothelial cells, and osteoblastic progenitor cells can promote the proliferation and differentiation of PDLSCs [67–69]. Bladder epithelial stem cells have multilineage potential and can effectively alleviate severe hindlimb ischaemia through paracrine action [70]. Taking advantage of paracrine signalling, the osteogenic differentiation of PDLSC sheets in vitro and in vivo was improved by co-culture with bladder epithelial stem cells [71]. Type IV collagen A2 (COL4A2) is one of the major structural components of the basement membrane, it can bind to bone morphogenetic proteins involved in angiogenesis and osteogenesis (such as osteogenin and osteogenic protein-1). The binding protein is presented in an immobilized form to stem cells and osteoprogenitor-like cells to initiate osteogenesis. When PDLSCs were cultured on decellularized BMSCs ECM (B-dECM) and decellularized PDLSCs ECM (P-dECM), PDLSCs showed stronger proliferation, stemness and osteogenic differentiation abilities. Additionally, the upregulation of COL4A2 in P-dECM inhibited the Wnt pathway and promoted osteogenic differentiation of PDLSCs [72]. Bone regeneration is a more complicated process than osteogenesis and physical function recovery. With the constant development of the PDLSC sheets for promoting bone formation, genetic modification may become another promising method at the same time [73]. In short, how to better improve the function of the sheets is still a problem worthy of in-depth study.

### Corneal regeneration of PDLSC sheets

DPSC sheets were shown to have the potential to promote corneal injury regeneration, analogously, PDLSC sheets showed regenerative effects on corneas. The cornea is a protruding structure in which cells are subjected to dome-shaped strain. In vitro, the dome-shaped mechanical stimulation can promote the multilayer, transparent structure of PDLSC sheets and the expression of corneal stroma markers with keratocyte inducing medium synergically. Tendon is very similar to the corneal stroma in structure, composition and gene expression profile, and significantly upregulation of tendon related genes was observed when BMSCs were treated with similar mechanical and chemical stimulation. In the future, more effective ways alone or in combination with chemical stimulation can be found by refining the mechanical stress to promote better corneal regeneration [74].

The current conventional method of sheets harvest requires approximately 10 days of culture, which is a barrier to clinical application. To address this problem, an appropriate method should be selected to store the obtained cell sheets while ensuring that its structural integrity and biological activity are not damaged. Cryopreservation has become a storage method for a variety of biological materials. By comparing the PDLSC sheets that had been cryopreservated for 3 months with the freshly prepared cell sheets, it was found that there was no significant difference in the cell proliferation rate, cell activity or karyotype analysis between the two types. The bone-like matrix formed in the body showed a network of type I and III collagen that were closely connected with each other, indicating that cryopreservation is a reliable treatment to preserve PDLSC sheets [75]. Nonetheless, whether longer treatment would cause damage to the biological activity and function of the cell sheets calls for further investigation.

Studies about regeneration of PDLSC sheets involving dental tissue, bone tissue and cornea will also have a positive impact on regeneration therapy of other tissues in the future. PDLSC sheets show better regeneration effects in periodontal tissue when compared to other tissues.

## Other DMSC sheets applications

**Fig. 3:**
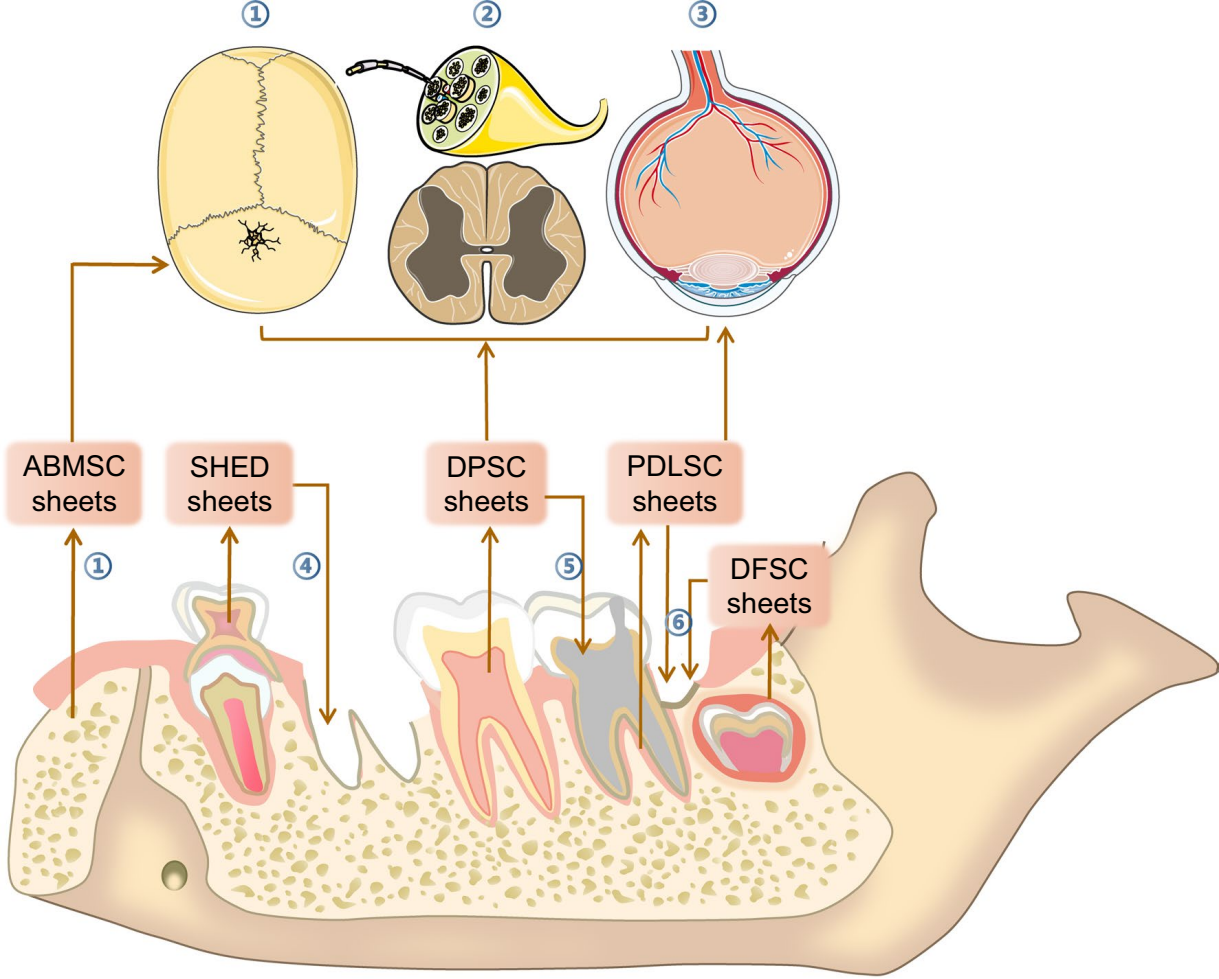
DMSC sheets in different tissues regeneration. DPSC sheets application dental tissue, bone, nervous system and cornea regeneration; PDLSC sheets application dental tissue, bone and cornea regeneration; SHED sheets application dental root regeneration; DFSC sheets application periodontal tissue regeneration; ABMSC sheets application bone regeneration. ① Bone; ② Nervous system; ③ Cornea; ④ Root; ⑤ Dental pulp; ⑥ periodontal tissue. The elements of bone, nervous system and cornea were obtained from the Smart Servier website: https://smart.servier.com/

In addition to DPSC sheets and PDLSC sheets, which have been widely applied to various regenerative therapies, other dental stem cell sheets currently used in regenerative medicine include DFSC, ABMSC and SHED sheets (Fig. 3 and Table 1). These cell sheets have been applied to dental tissue regeneration, but the applications of other diseases or tissue defects need further exploration.

**Table 1:** Regeneration of DMSC sheets in different study models

Dental stem cells	Tissue regeneration	Study model	Effect	Additional treatment	Cell sheet pretreatment time	Treatment time	References
DPSCs	Dental tissue	Immunodeficient mice subcutaneous space	Pulp-like tissue was observed in root canal	Rod-like structure based on single layer cell sheet	4 days	6 weeks	Itoh et al. [27]
		Swine experimental periodontal bone defects	Alveolar bone and soft tissue recovered significantly	–	10–15 days	12 weeks	Hu et al. [29]
		Root	Periodontal tissue-like fibres, dentin formation	TDM	–	12 weeks	Meng et al. [32]
		Immunodeficient mice subcutaneous	Higher blood-perfused vessel density	Recellularization and decellularization	14 days	30 days	Alghutaimel et al. [33]
		Beagle dogs root canal	Neovascularization, formation of dental Pulp-like tissues and mineralized structures	Decellularization	–	8 weeks	Alqahtani et al. [34]
	Bone	Mouse calvarial defects	Bone regeneration, cell sheet was in situ whole period	TH	14 days	8 weeks	Fujii et al. [35]
		Mouse calvarial defects	New bone formation, osteogenic differentiation in vitro	TH	14 days	8 weeks	Sato et al. [36]
		In vitro	More osteoblasts, bone matrix protein and calcified matrix moved to bone defect	Spherical tissue-engineered structure	5 weeks	–	Tatsuhiro et al. [38]
	Facial nerve	Rat facial nerve crush injury	Promoted axon extension, enhanced nerve regeneration	–	10–12 days	3 weeks	Ahmed et al. [40]
	Corneal	Rabbits LSCD	Functional regeneration and transparency of damaged corneas; expressed limbal stem cell markers	–	3 days	3 months	Gomes et al. [46]
PDLSCs	Periodontal tissue	Sheep mandible periodontal defects	Alveolar bone, thin cementum and periodontal fibres regeneration	Biphasic scaffold	7 days	5 and 10 weeks	Vaquette et al. [50]
		Immunodeficient mice dorsal surface	PDL-like dense collagen fibres, vascular regeneration	Titanium rod	6–10 days	8 weeks	Gao et al. [54]
		Immunodeficient mice subcutaneous implantation	PDL-like fibrous structure and mineralized tissue formation	Self-form cylindrical structure	7/14 days	4 weeks	Basu et al. [55]
		Nude mice subcutaneous	Cementum-like mineralized tissue and vertical or oblique insertion of collagen fibre onto the MBCP structure surface	rhBMP-2	14 days	4 weeks	Park et al. [57]
		Beagle dogs mandibular bone defect	PDL-like fibrous tissue was observed on the surface of the implant	Titanium rod	10 days	6 weeks	Washio et al. [58]
	Bone	Nude mice subcutaneous	Promote PDLSC proliferation, induce osteogenic differentiation and bone-like tissues	PRF	14 days	8 weeks	Wang et al. [60]
		Nude mice dorsa	Osteogenic related genes and proteins significantly improved; more bone formation	Osthole	10 days	4 weeks	Gao et al. [63]
		Nude mice dorsa	More new bone formation	AuNPs	7 days	4/8 weeks	Zhang et al. [65]
		Nude mice subcutaneous	Osteogenic differentiation was improved	Indirectly cocultured	10 days	6 weeks	Wu et al. [67]
		Rat maxillary defects and immunocompromised mice subcutaneous	More COL4A2 expression, more compact, thicker newly formed bone, thicker and denser collagen fibres	PDLSC cultured on B-dECM or P-dECM	10 days	8 weeks	Wen et al. [72]
	Corneal	In vitro	Promote PDLSC differentiated into keratinocytes	Mechanical stimulation	12 days	–	Li et al. [75]
DFSCs	Periodontal tissue	Rat omenta	Cementum and periodontal ligament-like tissue regeneration	Co-cultured with HERS	3 weeks	5 weeks	Bai et al. [78]
		Nude mice under the renal capsule	Periodontal ligament, cementum and alveolar bone regeneration	DDM	9–11 days	4 and 8 weeks	Feng et al. [80]
		Rat calvarial bone defect	New bone, periodontal-like tissues formation	–	2 weeks	2, 4, and 8 weeks	Yang et al. [81]
ABMSCs	Bone	Rabbit bone defect	Bone-like tissue formation	–	10 days	4 weeks	Liu et al. [84]
SHED	Root	Rats jaw bone defect	ALP, OCN and BSP expression were up-regulated, dentin and periodontal tissue regeneration	–	14 days	8 weeks	Yang et al. [85]

*ABMSCs* alveolar bone-derived mesenchymal stem cells, *ALP* alkaline phosphatase, *AuNPs* gold nanoparticles, *B-dECM* decellularized bone marrow mesenchymal stem cell extracellular matrix, *BSP* bone sialoprotein, *COL4A2* type IV collagen A2, *DDM* decalcified dentin matrix, *DFSCs* dental follicle stem cells, *DPSCs* dental pulp stem cells, *LSCD* limbal stem cell deficiency, *MBCP* micro-/macro-porous biphasic calcium phosphate, *OCN* osteocalcin, *P-dECM* decellularized periodontal ligament stem cells extracellular matrix, *PDL* periodontal ligament, *PDLSCs* periodontal ligament stem cells, *PRF* platelet-rich fibrin, *SHED* stem cells from human exfoliated deciduous teeth, *TDM*-treated dentin matrix, *TH* 4-(4-Methoxyphenyl) pyrido [40,30:4,5] thieno [2,3-b] pyridine-2-carboxamide

DFSCs express a higher level of colony-forming ability and osteogenic-related markers such as RUNX2, ALP and DSPP than DPSCs and PDLSCs [9]. Hertwig's epithelial root sheath (HERS) cells play an important role in the development of the root by secreting proteins related to tooth development, guiding DFSCs attachment to the newly formed root dentin [76] and acting as the signal centre for the development of the root [77]. Based on the above characteristics, sheets harvested from DFSCs co-cultured with HERS can form cementum and periodontal ligament-like tissue in vivo [78]. Although DFSCs and PDLSCs share similar mesenchymal origins, their respective cell sheets have different characteristics in the inflammatory microenvironment. In the experimental animal model of periodontitis, the bone regeneration in DFSC sheets group was more than PDLSCs group and was basically similar to normal bone tissue. Although the expression of osteogenic genes in PDLSC sheets were superior to that in DFSC sheets, the opposite result was shown in the inflammatory state. This may be the result of embryonic characteristics of DFSCs providing a favourable stem cell niche for periodontal regeneration [79]. In the process of periodontal regeneration, a certain space is needed. DFSC sheets were combined with decalcified dentin matrix (DDM) and hydroxyapatite/tricalcium phosphate to form a composite structure. It had a certain support strength and maintained a certain space during periodontal regeneration. Stem cells in DFSC sheets are conducive to long-term stable microvascular growth and blood supply, as well as to periodontal ligament, cementum and alveolar bone regeneration [80]. DFSC sheets combined with treated dentin matrix particles (TDMP) have similar regeneration effects [81].

BMSC sheets have been verified to be effective in bone regeneration in previous studies [82], but the application of BMSCs has many limitations, such as the invasive and painful process of collecting samples from the iliac crest, inflammatory stimulation and impairment in differentiation and stemness due to the age of the donor [83]. Compared with iliac-derived MSCs (Lon-BMSCs), the acquiring procedure of ABMSCs shows less trauma, more proliferation and osteogenesis abilities. ABMSC sheets had higher osteogenic differentiation and bone defect reconstruction ability than Lon-BMSCs sheets in the rabbit bone defect model after 4 weeks of treatment, implying, the degree of recovery of osteogenic-related genes and bone defect area was higher. Moreover, T helper 1 (Th1), type 1 macrophages (M1) and type 2 macrophages (M2) had no significant difference in different groups, indicating that the immunoregulation function of Lon-BMSCs and ABMSCs had no differentiation. ABMSCs exhibited immunosuppressive effects on monocyte activation and T cell activation and proliferation similar to Lon-BMSCs, both of them drove macrophages into an anti-inflammatory M2 phenotype [84].

SHED is another important type of DMSCs that is much younger than DPSCs and is predicted to possess the potential to regenerate periodontal tissues. To explore the potential of SHED in root regeneration, SHED sheets were grafted with TDM into the subcutaneous and jawbone of nude mice and rats. The osteogenic and odontogenic genes and proteins were upregulated after 14 days of induction, and dentin and periodontal tissue regeneration were also observed. Therefore, SHED sheets are promising materials that can be applied for biological root construction therapy and clinical applications. The remarkable protein synthesis and secretion functions of SHED can form a local microenvironment conducive to tissue repair and regeneration. L-Ascorbic acid, a component of cell sheets formation medium and osteogenic induction medium, is beneficial to the autocrine and paracrine functions of cells and promote the synthesis and accumulation of ECM, which may be a key factor in promoting a beneficial microenvironment [85]. SCAP sheets, from the DMSC sheets category, have the highest mineral tissue formation, being suitable for mineralized tissue formation [26]. Although the above cell sheets show multidirectional differentiation potential, DPSCs, PDLSCs and SCAPs showed no significant difference regarding tissue structure and ECM protein expression. They have unique features and work on various tissue regeneration differently. In the future, merits and demerits of different DMSC sheets can be further investigated, as well as their application.

Cell sheet technology has made remarkable progress since its first invention. Nevertheless, the applications of this technology are still in their infancy, and shortcomings should not be ignored. The following are problems during cell sheets culture. First of all, DMSC sheet culture generally takes about 14 days which is not suitable for urgent use. Secondly, the quality of cell sheets obtained from different batches of cell sources varies widely and the high cost of the culture process makes mass production hard. Besides, personalized and accurate repair is impossible with current technology, but the combination of cell sheets and 3D printing technology is likely to be a potential solution [37]. On the other hand, the transplantation and transport of single-cell sheet mechanical property is also worth noticing. Simple cell sheets transplantation usually does not generate blood vessels by themselves, and multilayer cell sheets applied to large or deep tissue defects may be difficult to ensure nutritional supply after transplantation with a possible affected cell viability. Moreover, the infection risks, trauma from transplantation, immune-related rejection, oncogenicity and other potential side effects should not be ignored. In order to minimize the damage and avoid postoperative infection, it is recommended to follow the transplant instructions strictly, use minimally invasive methods and perform aseptic practice all the time. Generally, autologous cells are characterized by an extremely low probability of triggering an immune response, but unfortunately autologous cells are very limited and are subjected to donors' physical condition [16, 86]. The usage of immunosuppressant or decellularization of allogeneic cell sheets may be required to reduce immune response if necessary. Limited research has shown that the high-density cellular interconnection structure of the cell sheets can directly promote the regeneration of target tissues, and the underlying mechanisms have not been clarified. The dynamics of cell migration from the cell sheets and its role in tissue formation in vivo, the integration of the cell sheets in vivo and the extent to which cells contribute directly to regeneration are needed to be unfold [87]. As for practical application, scientific supervision systems and standardized training for relevant practitioners is inevitable. Currently in Japan, relevant laws and regulations have been issued [88], which has set up a great example for other countries and regions.

## Conclusions

Current studies have confirmed that a variety of DMSC sheets could promote the regeneration of abundant tissues, showing vigorous regeneration and repair potential in dental tissue, bone, nerve and corneal regeneration. Although the regenerative effect of DMSC sheets is encouraging, not all DMSC sheets can promote tissue regeneration in vivo, the way of choosing the suitable seed cells correctly is essential. Additionally, the DMSC sheets themselves also need more reform. Some improvements, such as combining them with drugs or bioactive molecules, or integrated with scaffold, are necessary. The reformed DMSC sheets are expected to manage cell metabolism or biological reactions in the body. Currently, the technology and application of cell sheets are basically limited to the vitro histology and animal models and few clinical trials, more complicated clinical situations need to be considered to transform them into widespread clinical applications. Moreover, the mass production and long-term effective preservation method and trauma remain unresolved. Moreover, existing researches have not deeply discussed the mechanism and the role between specific components in cell sheets and tissue regeneration needs to further explode.

## Data Availability

Not applicable.

## Abbreviations

ABMSCs: Alveolar bone-derived mesenchymal stem cells
ADSCs: Adipose-derived stem cells
ALP: Alkaline phosphatase
AuNPs: Gold nanoparticles
B-dECM: Decellularized bone marrow mesenchymal stem cell ECM
BDNF: Brain-derived neurotrophic factor
BMSCs: Bone marrow mesenchymal stem cells
BSP: Bone sialoprotein
COL 1: Collagen type 1
COL4A2: Type IV collagen A2
CSM: Composite membrane
dECM: Decellularized extracellular matrix
DDM: Decalcified dentin matrix
DFSCs: Dental follicle stem cells
DMP-1: Dentin matrix protein 1
DMSCs: Dental-derived mesenchymal stem cells
DPSCs: Dental pulp stem cells
DSPP: Dentin sialophosphoprotein
ECM: Extracellular matrix
ERK: Extracellular signal-regulated kinase
GDNF: Glial cell-derived neurotrophic factor
GMSCs: Gingival mesenchymal stem cells
HERS: Hertwig’s epithelial root sheath
HGF: Hepatocyte growth factor
HP: Heparin-poloxamer
LED: Light-emitting diode
Lon-BMSC: Iliac-derived MSC
LSCD: Limbal stem cell deficiency
MBCP: Micro-/macro-porous biphasic calcium phosphate
M1: Type 1 macrophages
M2: Type 2 macrophages
MSCs: Mesenchymal stem cells
NT3: Neurotrophic factor-3
NTF: Neurotrophic factor
OCN: Osteocalcin
OPN: Osteopontin
PBMT: Photobiomodulation therapy
P-dECM: Decellularized PDLSC
PDL: Periodontal ligament
PDLSCs: Periodontal ligament stem cells
PRF: Platelet-rich fibrin
rhBMP-2: Recombinant human bone morphogenetic protein-2
ROS: Reactive oxygen species
RUNX2: Runt-related transcription factor 2
SCAPs: Stem cells from the apical papilla
SHED: Stem cells from human exfoliated deciduous teeth
TBP: Tributyl phosphate
TDM: Treated dentin matrix
TDMP: Treated dentin matrix particles
TGF-β: Transforming growth factor β
TGSCs: Tooth germ stem cells
TH: 4-(4-Methoxyphenyl)pyrido[40,30:4,5]thieno[2,3-b]pyridine-2-carboxamide
Th1: T helper 1
TRCD: Temperature-responsive polymer-grafted cell culture dishes
UC-MSC: Umbilical cord mesenchymal stem cell
VEGFR1: Vascular endothelial growth factor receptor 1
